# Immunoaging at Early Ages Could Drive a Higher Comorbidity Burden in People with HIV on Antiretroviral Therapy Compared with the Uninfected Population

**DOI:** 10.3390/ijms252010930

**Published:** 2024-10-11

**Authors:** Cora Loste, Macedonia Trigueros, Francisco Muñoz-López, Víctor Urrea, Ana Martínez, Sandra González, Jordi Puig, Marta Martín, Anna Bonjoch, Patricia Echeverría, Marta Massanella, Eugenia Negredo

**Affiliations:** 1 Department of Infectious Diseases, University Hospital Germans Trias i Pujol, Campus Can Ruti, 08916 Barcelona, Spain; 2 Fight Against Infections Foundation, Campus Can Ruti, 08916 Badalona, Spain; 3 Chair in Infectious Diseases and Immunity, Centre for Health and Social Care Research (CESS), Faculty of Medicine, University of Vic-Central University of Catalonia (UVic-UCC), 08500 Vic, Spain; 4 PhD Programme in Medicine and Biomedical Sciences, Doctoral School, University of Vic-Central University of Catalonia (UVic-UCC), C. Dr. Junyent, 1, 08500 Vic, Spain; 5 IrsiCaixa, 08916 Badalona, Spain; 6 Department of Medicine, Autonomous University of Barcelona, 08193 Barcelona, Spain; 7 Cell Biology Unit, Department of Cell Biology, Physiology and Immunology, Biosciences School, Universitat Autònoma de Barcelona, 08193 Bellaterra, Spain; 8 CIBER Infectious Diseases (CIBERINFEC), Institute of Health Carlos III (ISCIII), 28029 Madrid, Spain

**Keywords:** HIV, aging, inflammaging

## Abstract

This is an observational, cross-sectional, comparative case–control, pilot study aimed at assessing the impact of HIV infection and age on immunological markers in people with HIV (PWH) on antiretroviral therapy (ART). The study included 40 PWH on ART, divided into two age groups (40–45 years vs. ≥60 years), and 30 HIV-uninfected controls matched by sex and age. The results show that older PWH on ART had more comorbidities and a higher frequency of CD8 T cells compared to older controls, with a significant decrease in CD8 naïve T cells with age. Additionally, younger PWH on ART exhibited higher frequencies of activated CD8 T cells and elevated levels of inflammatory markers (sCD14, IL-6) compared to age-matched controls, with values similar to those of older PWH on ART. These findings suggest that younger PWH on ART may experience accelerated immunoaging, highlighting the need for early interventions in this population.

## 1. Introduction

Aging is a physiological process that affects all individuals and is associated with an increased likelihood of developing diseases due to the gradual loss of biological functions [1]. The immune system, like other organs, ages and develops an immunosenescent and proinflammatory (inflammaging) state that affects both innate (dendritic cells, natural killer cells, and monocytes) and adaptive (B and T cell) immune responses [2,3,4]. This state has a significant impact on the survival and fragility of the organism by increasing susceptibility to infectious diseases, cancer, and autoimmune phenomena, as well as decreasing vaccine efficacy [5].

On the other hand, chronic infections are thought to be the main drivers of accelerated immunosenescence, with HIV infection being a clear example [6,7,8,9]. On top of age-related immunosenescence, people with HIV (PWH) experience chronic immune activation, inflammation, and immune deregulation, leading to increased morbidity and mortality compared to the general population [4,10]. Even PWH on antiretroviral therapy (ART) who maintain sustained virological suppression present persistent low-grade inflammation. In addition, prolonged exposure to ART is another factor contributing to accelerated aging in PWH on ART, especially former ART recipients [11]. Finally, CMV co-infections or bacterial translocation are more common in PWH than in the general population, further promoting immune activation and systemic inflammation [7].

Advancements in ART have enabled many PWH on ART to live to older ages, particularly those in resource-rich areas. According to data from the Centers for Disease Control and Prevention (CDC), the threshold age for defining older adults in PWH is 50 years [10]. Predictive models suggest that by 2030, 70% of this population in the United States will be 50 years of age or older [12,13]. Subsequently, the HIV-related inflammation would be added to the natural process of inflammaging [3]. In order to assess the effect of age and HIV infection on the immunological aging process, we compared different immunological parameters of T cells (subset distribution, senescence, activation, and exhaustion) and inflammation between PWH on ART and HIV-uninfected people stratified by age (a younger vs. an older group). 

## 2. Results

**Figure 1:**
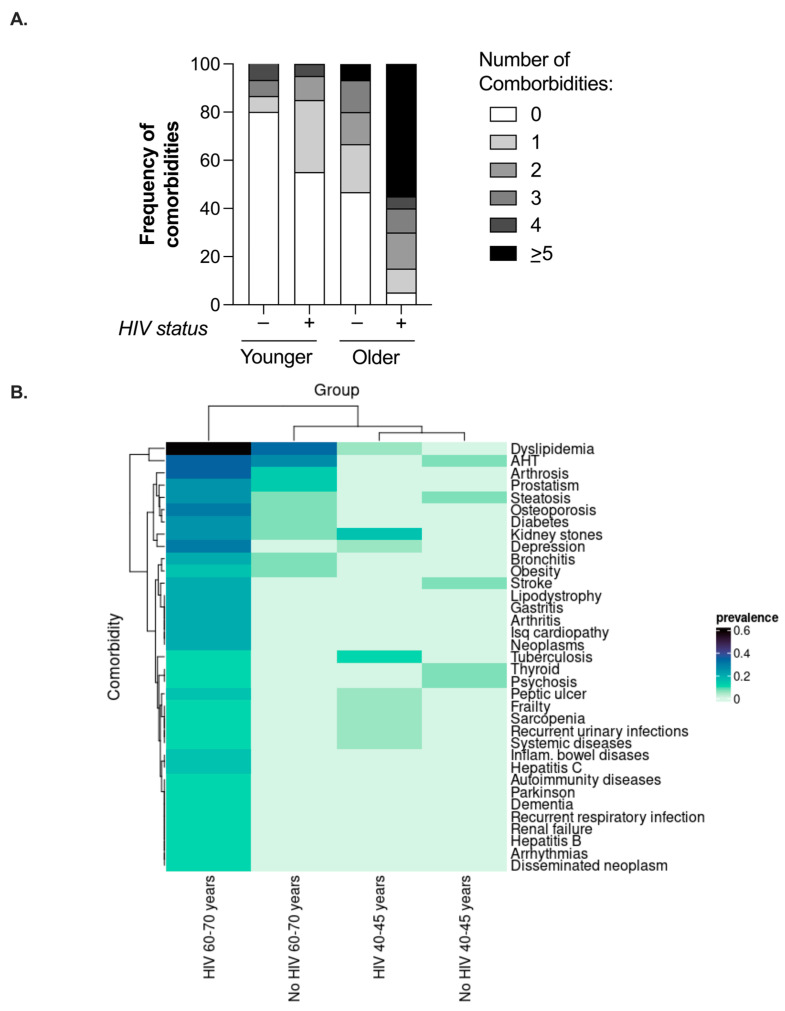
Comorbidities in the study groups. (**A**) Number of comorbidities according to age and HIV status. (**B**) A heat map representing the relative frequency of each comorbidity in each group.

Forty PWH on ART (20 in the older group and 20 in the younger group) matched by age and gender with 30 participants without HIV infection were included. The characteristics of the study participants are described in Table 1. Notably, the median number of comorbidities and the percentage of subjects with ≥5 comorbidities were higher in the older PWH on ART (Table 1 and Figure 1A); 55% of older PWH on ART had ≥5 comorbidities, while the corresponding figure was 7% among the uninfected controls (Table 1 and Figure 1A). Additionally, 80% of the younger uninfected controls did not show any comorbidity, while 55% of the younger PWH on ART were comorbidity-free (Figure 1A). The percentage of smokers and ex-smokers was 42.5% among the PWH on ART vs. 16.7% among the uninfected controls, reflecting a known tendency among PWH. The most frequent comorbidities in each group are shown in Figure 1B.

### 2.1. CD4 and CD8 T-Cell Distribution

**Figure 2:**
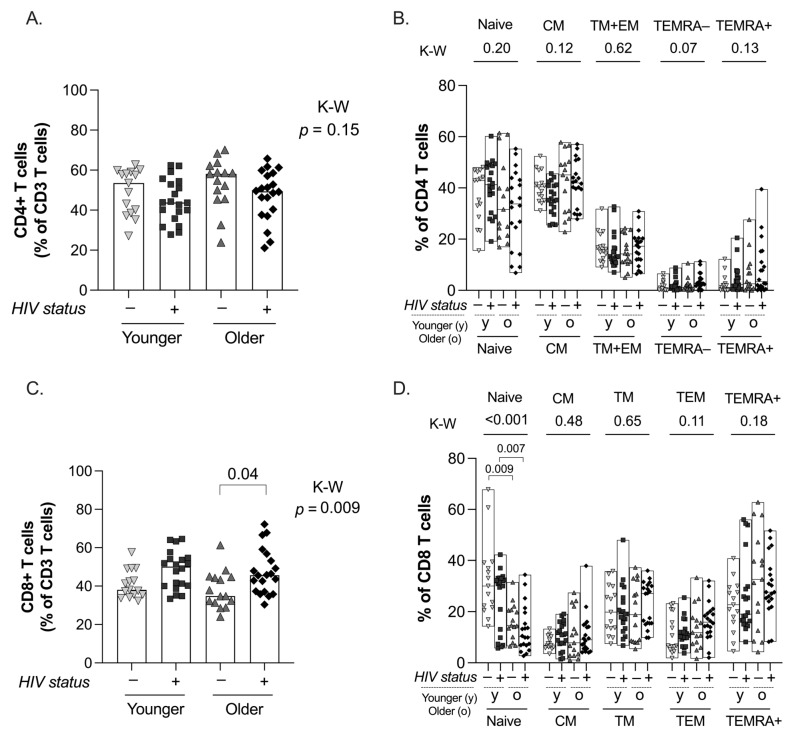
Total and CD4 and CD8 T-cell subsets. The frequency of total (**A**) and CD4 T-cell subsets (**B**) are plotted for the 4 groups under study. The frequency of total (**C**) and CD8 T-cell subsets (**D**) are shown for the 4 groups under study. For all panels, individual and median values are indicated for the 4 groups under study: younger uninfected (inverted triangles), younger PWH on ART (squares), older uninfected (triangles), and older PWH on ART (diamonds). *p*-values from Kruskal–Wallis tests are indicated for the comparison between the 4 groups under study, and only significant *p*-values from Dunn’s multiple comparison tests between groups are shown. For (**B**,**D**), Kruskal–Wallis tests were performed for each individual T-cell subset.

The frequency of CD4 T cells was similar between groups independent of age (*p* = 0.15 K-W test, Figure 2A). Regarding CD4 T-cell subset distribution, the frequency of TEMRA-CD4 T cells tended to be different between groups without reaching statistical significance (*p* = 0.07 K-W test, Figure 2B). The CD8 T-cell compartment showed more differences. As expected, the frequency of CD8 T cells was significantly different between groups (*p* = 0.009 K-W test, Figure 2C); in particular, older PWH on ART showed statistically higher frequencies compared to their uninfected counterparts (*p* = 0.04, Dunn’s comparison test). In addition, the frequency of CD8 naïve T cells was significantly different between study groups (Figure 2D; *p* < 0.001 K-W test), with lower frequencies in both older groups observed, regardless of HIV status (*p* = 0.007 and *p* = 0.009 between younger and older PWH on ART and control groups, respectively, Dunn’s comparison tests). In contrast, we did not observe any significant differences in the memory compartment according to age groups.

### 2.2. Senescence, Activation, and Exhaustion of T Cells

**Figure 3:**
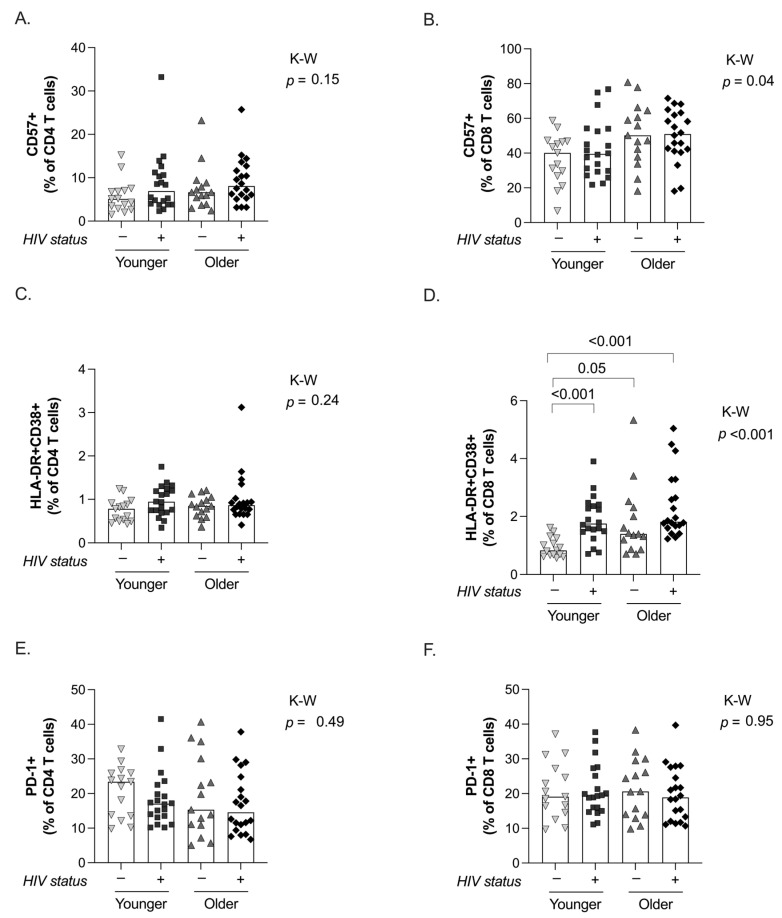
Immune senescence, activation, and exhaustion of CD4 and CD8 T cells. The frequency of senescent (CD57+) CD4 (**A**) and CD8 T cells (**B**) are plotted for the 4 groups under study. A comparison of the levels of immune activation, measured by the frequency of HLA-DR+CD38+ cells, is shown for CD4 (**C**) and CD8 T cells (**D**). Finally, the frequency of exhausted (PD-1+) CD4 (**E**) and CD8 T cells (**F**) is plotted. For all panels, individual and median values are indicated for the 4 groups under study: younger uninfected (inverted triangles), younger PWH on ART (squares), older uninfected (triangles), and older PWH on ART (diamonds). *p*-values from Kruskal–Wallis tests are indicated for the comparison among the 4 groups under study, and only significant *p*-values from Dunn’s multiples comparison tests between groups are shown.

CD57+ expression was examined as a marker of senescence in the total CD4 and CD8 T-cell populations. No differences were seen in the frequency of senescent CD4 T cells between all groups (*p* = 0.15 K-W test, Figure 3A). Conversely, we observed significant differences in CD57+ expression in total CD8 T cells across all groups (*p* = 0.04 K-W test, Figure 3B).

Activation of the immune system in T-cell subpopulations was assessed for co-expression of the HLA-DR and CD38 markers. While we did not observe differences in the frequency of activated CD4 T cells (Figure 3C), we found significant differences in the CD8 T-cell compartment (*p* < 0.001 K-W test, Figure 3D). In particular, younger uninfected controls showed a significantly lower frequency of activated CD8 T cells than younger PWH on ART and older groups (*p* < 0.05, in all cases, Dunn’s comparison test, Figure 3D). Importantly, the levels of activated CD8 T cells in the younger PWH on ART group were comparable to those found in both older groups and no differences were observed between the older groups (Figure 3D).

Finally, we analyzed the expression of PD-1 in T cells, a well-characterized marker of cell exhaustion. No differences were observed in the expression of PD-1 in total CD4 or CD8 T cells among groups (Figure 3E and 3F, respectively).

### 2.3. Proinflammatory Status

**Figure 4:**
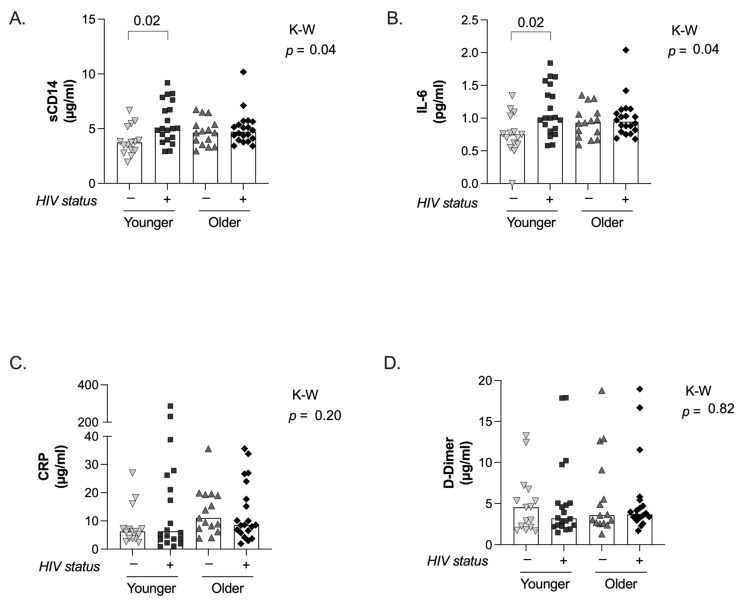
**Soluble proinflammatory markers.** The levels of soluble CD14 (sCD14, (**A**)), IL-6 (**B**), C-reactive protein (CRP; (**C**)), and D-Dimer (**D**) are plotted for the 4 groups under study. For all panels, individual and median values are indicated for the 4 groups under study: younger uninfected (inverted triangles), younger PWH on ART (squares), older uninfected (triangles), and older PWH on ART (diamonds). *p*-values from Kruskal–Wallis tests are indicated for the comparison among the 4 groups under study, and only significant *p*-values from Dunn’s multiples comparison tests between groups are shown.

Statistically significant differences between groups in sCD14 and IL-6 were seen (*p* = 0.04 K-W test in both cases, Figure 4A,B), being the levels of both soluble markers significantly higher in the younger PWH on ART group compared to the younger controls (*p* = 0.02 Dunn’s correction tests, in both cases). The levels of those markers in the younger PWH on ART group were comparable to those found in both older groups. We did not find differences between the groups in the plasmatic levels of CRP and D-dimer (Figure 4C,D).

### 2.4. Contribution of HIV Infection and Age in Immunoaging

To further investigate the association between HIV infection and age to T-cell status and inflammatory processes, we performed a one-way ANOVA screening to determine the significant variants. Like our previous analysis, this approach revealed that the number of comorbidities, the frequency of total and naïve CD8 T cells, the frequency of senescent (CD57+), and activated (HLA-DR+CD38+) CD8 T cells, as well as the soluble markers sCD14 and IL-6, were different between both groups of age and the HIV infection status (p-Adj < 0.1 in all cases, Table 2). Importantly, no differences were observed in the CD4 T-cell compartment. Then, we performed a two-way ANOVA to assess the independent contribution of age and HIV infection factors and their interrelationship for each selected variable (Table 2). The number of comorbidities was significantly dependent on HIV infection and age (*p* < 0.001 in both cases), and there was an interaction between both variables (*p* < 0.001). In contrast, the frequency of CD8 T cells was only significantly associated with HIV infection (*p* ≤ 0.001), while the percentage of naïve CD8 T cells was linked to HIV infection (*p* = 0.03) and age (*p* < 0.001), without interaction between factors. The senescence of CD8 T cells (CD57+) was only significantly associated with age (*p* = 0.008), and the activation of the CD8 T-cell subset (HLA-DR+CD38+) was associated with both HIV infection (*p* ≤ 0.001) and age (*p* = 0.001). However, no interaction between both factors was observed. Finally, both soluble markers, sCD14 and IL-6, were significantly associated with HIV infection (*p* = 0.01 and *p* = 0.008, respectively) and no association was found with age.

## 3. Discussion 

Inflammaging and HIV-related inflammation could prematurely coexist in older PWH on ART. In our cohort, differences in some senescence and inflammatory markers were observed between PWH on ART and uninfected younger groups, suggesting a premature immunoaging in PWH on ART. The frequency on senescent CD8 T cells was only associated with age, while the frequency of CD8 T cells and the inflammatory markers sCD14 and IL-6 were associated with HIV infection.

Studies focused on PWH have shown signs of accelerated immune senescence, particularly in CD8 T-cell subsets [14]. The alterations in lymphocyte populations linked to the aging process in general population mirror those observed in HIV infection at younger ages [15]. PWH, even those with viral suppression, exhibit higher levels of immune activation and inflammation markers compared to HIV-uninfected individuals [6,15,16]. However, a recent extensive study involving 111 PWH on ART and 114 controls concluded that HIV and age do not synergistically affect age-related T-cell markers [17]. Our study, which examined a broader number of markers of T-cell subsets and compared younger and older individuals, supports these findings; although age and HIV infection impact on immunological parameters, no synergistic effect was seen between both factors.

Regarding T-cell subpopulations, as expected, we observed lower percentages of naïve CD8 T cells in older participants compared to younger individuals in both groups (HIV vs. uninfected groups), with significant differences in certain subsets between PWH on ART and controls.

According to other studies, our results support the fact that levels of some immunological parameters are higher at younger ages in PWH on ART [11,16,17,18,19] compared to their controls counterparts. In our specific case, we observed that the increased levels of the soluble markers sCD14 and IL-6 were associated with HIV infection (Table 2). In addition, the younger PWH on ART group showed a higher frequency of the activation marker HLA-DR+CD38+ on CD8 T cells, being similar to those observed in older participants. Interestingly, this marker was associated with HIV infection and age (Table 2). However, no significant differences were found in exhaustion parameters.

In terms of classic inflammatory biomarkers, there is much evidence that some of these markers are increased in PWH [20,21,22,23,24,25], with some associations with clinical consequences. The SMART study found that higher levels of IL-6, CRP, and D-dimer are associated with an increased risk of cardiovascular diseases in PWH, as an independent factor [20,21]. Another study showed increased levels of proinflammatory cytokines, such as IL-6 [22]. However, the most consistently elevated marker among PWH on ART is sCD14, a surrogate marker of microbial translocation [19,22,23,25]. Indeed, some studies have already suggested that a persistent elevation of sCD14 in PWH on ART is associated with all-cause mortality, and that could be a predictive biomarker for chronic diseases in this population [22,24]. Our study found higher levels of sCD14 and IL-6 in the younger PWH on ART group compared to the younger controls and similar to those seen in the older groups, indicating an advanced inflammation. The increased levels of these markers were mainly associated with HIV infection.

The advanced inflammatory state observed among our PWH on ART could potentially accelerate the aging of the immune system and lead to long-term clinical consequences. In fact, the older PWH on ART group had a significantly higher burden of comorbidities compared to older controls, similarly to our previous results [26]. Therefore, early interventions should be implemented at early ages. The use of statins to decrease the chronic inflammation in PWH on ART with a moderate/low cardiovascular risk has shown a 35% reduction in the risk of cardiovascular event occurrence compared to the control group in the REPRIEVE study [27]. Interventions like this must be carried out at early ages to delay or avoid comorbidities.

The main limitations of our study are the small sample size and the cross-sectional analysis of data, which prevent us from evaluating changes over time. It is also worth noting that, although the percentage of smokers/ex-smokers was higher among PWH, it is well-know that HIV infection itself is an independent risk factor for comorbidities and other age-related factors [26]. Additionally, few markers of inflammation and activation were assessed since the study was mainly focused on T cells. Conversely, the act of assessing two different age ranges in both populations sheds some light to the question of the effect of HIV and age on the aging of the immune system.

## 4. Materials and Methods

### 4.1. Study Design and Study Participants

This is a study based on a case–control series including PWH on ART in two age ranges (younger: 40–45 years old vs. older: ≥60 years old) and HIV-uninfected controls matched by sex and age (±2 years).

For the HIV group, we included participants with chronic HIV infection for at least 8 years, a virological suppression for at least 5 years, and being on stable ART with regimens including Darunavir, Elvitegravir, Raltegravir, or Dolutegravir, with one or two nucleosides. A nadir CD4 T-cell count of less than 200 cells/µL was an exclusion criterion. A total of 40 PWH on ART were enrolled, 20 participants in the younger group (40–45 years old) and 20 individuals in the older group (≥60 years old). Thirty matched HIV-uninfected participants were included as a control group (N = 15 in the younger group and N = 15 in the older group). For both groups, people with active diseases such as infections, including hepatitis B or C, immune disorders, or neoplasms were excluded.

### 4.2. Study Variables

Demographic and HIV-related data were collected from medical records to characterize our cohort. These data included sex, age, race, medical history (comorbidities, toxic habits, date of HIV diagnosis, nadir and current CD4 T-cell counts, current viral load, current ART, and concomitant treatments).

### 4.3. Sample Processing

A single blood sample was collected by venipuncture in heparin vacutainer tubes (BD Biosciences) from all participants and immediately processed for plasma and peripheral blood mononuclear cell (PBMC) isolation. The plasma was stored at −80 °C until use. The PBMCs were obtained from cell concentrates layered on Ficoll–Hypaque density gradients and were cryopreserved until use.

### 4.4. CD4 and CD8 T Cell Immunophenotyping

Frozen PBMCs were thawed at 37 °C, washed twice in RPMI/20% of fetal bovine serum (FBS) and incubated for 1 h at 37 °C before staining with an 11-color panel, including: CD3–BV605, CD4–FITC, CD8–BV510, CD45RA–AF700, CD197/CCR7–PE-CF594, CD28–PE, CD57–APC, CD279/PD-1–BV421, CD38–PerCP-Cy5.5, and HLA-DR–BV650 (Supplementary Table S1, all antibodies from BD Biosciences, San Jose, CA, USA). Cell viability was assessed using the LIVE/DEAD Fixable Near IR viability Kit (Life Technologies, Eugene, OR, USA). We evaluated the distribution of CD4 and CD8 T-cell differentiation subsets, including naïve, central memory, transitional memory, effector memory, and terminally differentiated (see the gating strategy in Supplementary Figure S1), and the frequency of activated (CD38 and HLA-DR), exhausted (PD-1), and immunosenescent (CD57) cells in T cells. Samples were acquired with a LSRFortessa flow cytometer (BD Bioscience, San Jose, CA, USA). Data were analyzed using FlowJo software V10.10 (Ashland, OR, USA). 

### 4.5. Quantification of Soluble Markers

Frozen plasma samples were thawed and assayed from the levels of different makers by ELISA using commercially available ELISA kits and following the manufacturer’s instructions: interleukin-6 (IL-6, IL-6 Human ELISA kit, High Sensitivity, from ThermoFisher, Vienna, Austria) and high-sensitivity C-reactive protein (CRP, Human CRP ELISA kit from RayBiotech Norcross, GA, USA) were used as inflammatory markers; D-dimer (Human D-Dimer ELISA kit, RayBiotech Norcross, GA, USA) as a coagulation marker; and soluble CD14 (sCD14, Human CD14 ELISA kit from Diaclone, Besançon, France ) as a marker of bacterial translocation.

### 4.6. Statistical Analysis:

Continuous variables were described using medians and the interquartile range (IQR, 25th and 75th percentiles), whereas categorical factors were reported as percentages. Quantitative variables were compared using the non-parametric Kruskal–Wallis (K-W) test, followed by Dunn’s test to identify differences between the four groups under study. To better understand the contribution of HIV infection, age, and/or both T-cell status and inflammatory processes, we used linear regression models. First, we performed the Box–Cox method to determine the most suitable transformation to fit our variables into a normal distribution. If the lambda coefficient from the Box–Cox calculation was equal to 1, the data were normally distributed, and no transformation was required. In contrast, if the lambda coefficient was close to 0, 0.5, or −0.5, we transformed the data to log10, square root, and inverse of square root, respectively. Then, an initial screening through linear regression models was performed, followed by FDR correction for statistical significance (adjusted *p*-value, p-Adj), to choose the variables affected either by HIV infection or age. Second, we carried out a two-way ANOVA test with markers that showed p-Adj < 0.1, which gave us information about the effects of HIV infection and age independently, and the interaction of both. Analyses were performed with Prism 9.1.2 (GraphPad, Version 10.2.2 (341), 19 March 2024, Boston, MA, USA) and RStudio (Software 2022.02.01). For the tests carried out in this study, a significance level of 0.05 was defined, which corresponds to a confidence level of 95%.

## 5. Conclusions

In conclusion, our results support that HIV infection has a negative impact on inflammation and activation markers, while age affects mainly immunosenescence, specifically in CD8 T cells. The detection of higher levels in some biomarkers in the younger PWH on ART group suggests that aging-related changes occur earlier in this population at the immunological level (accelerated immunological aging), driving the emergence of comorbidities.

Early intervention in young PWH on ART focused on reducing this inflammatory state and, consequently, the long-term comorbidities should be implemented.

## Figures and Tables

**Table 1:** Participant characteristics.

	PWH on ART		Uninfected	
	Younger n = 20	Older n = 20	Younger n = 15	Older n = 15
**Demographic data**				
Age, years, median [IQR]	43 [42–44]	63 [61–67]	42 [41–44]	63 [62–67]
Sex, male, n (%)	15 (75)	18 (90)	10 (67)	14 (93)
Race, Caucasian, n (%)	17 (85)	19 (95)	15 (100)	15 (100)
Toxic Habits				
Smoker, n (%)	8 (40)	0	0	1 (6.7)
Ex-smoker, n (%)	2 (10)	7 (35)	2 (13.3)	1 (6.7)
Active use of alcohol, n (%)	0	1 (5)	0	0
Comorbidities				
Number of comorbidities, median [IQR]	0 [0,1]	5 [2–6]	0 [0]	1 [0–2]
With ≥5 comorbidities, n (%)	0 (0)	11 (55)	0 (0)	1 (7)
HIV-related information				
>8 years from diagnosis, n (%)	20 (100)	20 (100)	-	-
Nadir CD4 T cell, median [IQR]	380 [328–451]	413 [334–476]	-	-
Nadir CD4 T-cell count <200 cells/µL, n (%)	0 (0)	0 (0)	-	-
Current CD4 T-cell count, cells/µL, median [IQR]	842 [752–995]	822 [685–953]	-	-
VL undetectable (<20 copies/mL), n (%)	20 (100)	20 (100)		
Current ART treatment, n (%)				
INSTI-bases	13 (65)	13 (65)	-	-
PI-based	5 (25)	3 (15)	-	-
Combination INSTI/NNRTI-based	1 (5)	2 (10)	-	-
Combination INSTI/PI-based	1 (5)	2 (10)	-	-

PWH: people with HIV; ART: antiretroviral treatment; VL: viral load; INSTI: integrase strand transfer inhibitors; PI: protease inhibitor; NNRTI: non-nucleoside reverse transcriptase inhibitor.

**Table 2:** Contribution of HIV infection and age in immunoaging.

	Linear Models		ANOVA Two-Way		
	Significance by Age or Infection	Adjusted *p*-Values (p-Adj)	HIV Infection Term (p-Adj)	Age Term (p-Adj)	Interaction HIV and Age (p-Adj)
Number of comorbidities	<0.001	<0.001	<0.001	<0.001	<0.001
CD4 T cells					
% CD4+	0.09	0.15			
Naive (% CD4 T cells)	0.19	0.25			
Central memory (% CD4 T cells)	0.06	0.16			
TM+EM (% CD4 T cells) ^1^	0.94	0.94			
TEMRA− -(% CD4 T cells) ^1^	0.06	0.16			
TEMRA+ (% CD4 T cells) ^1^	0.07	0.16			
CD57+ (% CD4 T cells) ^1^	0.06	0.16			
HLA-DR+CD38+ (% CD4 T cells) ^1^	0.11	0.17			
PD-1+ (% CD4 T cells) ^1^	0.30	0.37			
CD8 T cells					
% CD8+ ^2^	0.001	0.008	<0.001	0.32	0.56
Naive (% CD8 T cells) ^2^	<0.001	<0.001	0.03	<0.001	0.68
CM (% CD8 T cells) ^2^	0.49	0.58			
TM (% CD8 T cells) ^1^	0.57	0.63			
EM (% CD8 T cells) ^2^	0.07	0.16			
TEMRA+ (% CD8 T cells) ^2^	0.19	0.26			
Replicative senescence (% of CD8 T cells) ^2^	0.09	0.16			
CD57+ (% CD8 T cells)	0.02	0.09	0.54	0.008	0.38
HLA-DR+CD38+ (% CD8 T cells) ^1^	<0.001	<0.001	<0.001	0.001	0.14
PD-1+ (% CD8 T cells) ^1^	0.93	0.94			
Soluble Markers					
Soluble CD14 ^1^	0.04	0.08	0.01	0.91	0.08
IL-6 ^1^	0.03	0.08	0.008	0.81	0.06
CRP ^3^	0.11	0.15			
D-dimer ^3^	0.9	0.9			

^1^ Log10-transformed,^2^ squared root-transformed and ^3^ inverse of square root-transformed. CM: central memory; TM: transitional memory; EM: effector memory; TEMRA+: effector memory CD45RA+; sCD14: soluble CD14; IL-6: interleukin 6; CRP: C-reactive protein; p-Adj: *p*-value adjusted.

## Data Availability

The data will be accessible from fr corresponding author (CL) upon request.

## Supplementary Materials

The following supporting information can be downloaded at: https://www.mdpi.com/article/10.3390/ijms252010930/s1.
