# Supplementary material for: Immunoaging at Early Ages Could Drive a Higher Comorbidity Burden in People with HIV on Antiretroviral Therapy Compared with the Uninfected Population
Source: Int J Mol Sci. 2024 Oct 11;25(20):10930. doi: 10.3390/ijms252010930 (PMC11507496; doi:10.3390/ijms252010930)
Supplement: Supplementary file 1 [file ijms-25-10930-s001.zip › Supplementary Table s1.pdf]

Supplementary table S1:

Antibodies	Clone	Brand
CD3 BV605	Sk7	BD
CD4 FITC	RPA-T4	BD
CD8 BV510	SK1	BD
CD45RA AF700	HI100	BD
CD197 PE-CF594	150503	BD
CD279 BV421	EH12.1	BD
CD57 APC	NK-1	BD
CD38 PerCP-Cy 5.5	HIT2	BD
HLA-DR BV650	G46-6	BD
CD28 PE		BD
