# Supplementary figures and images for: Immunoaging at Early Ages Could Drive a Higher Comorbidity Burden in People with HIV on Antiretroviral Therapy Compared with the Uninfected Population
Source: Int J Mol Sci. 2024 Oct 11;25(20):10930. doi: 10.3390/ijms252010930 (PMC11507496; doi:10.3390/ijms252010930)

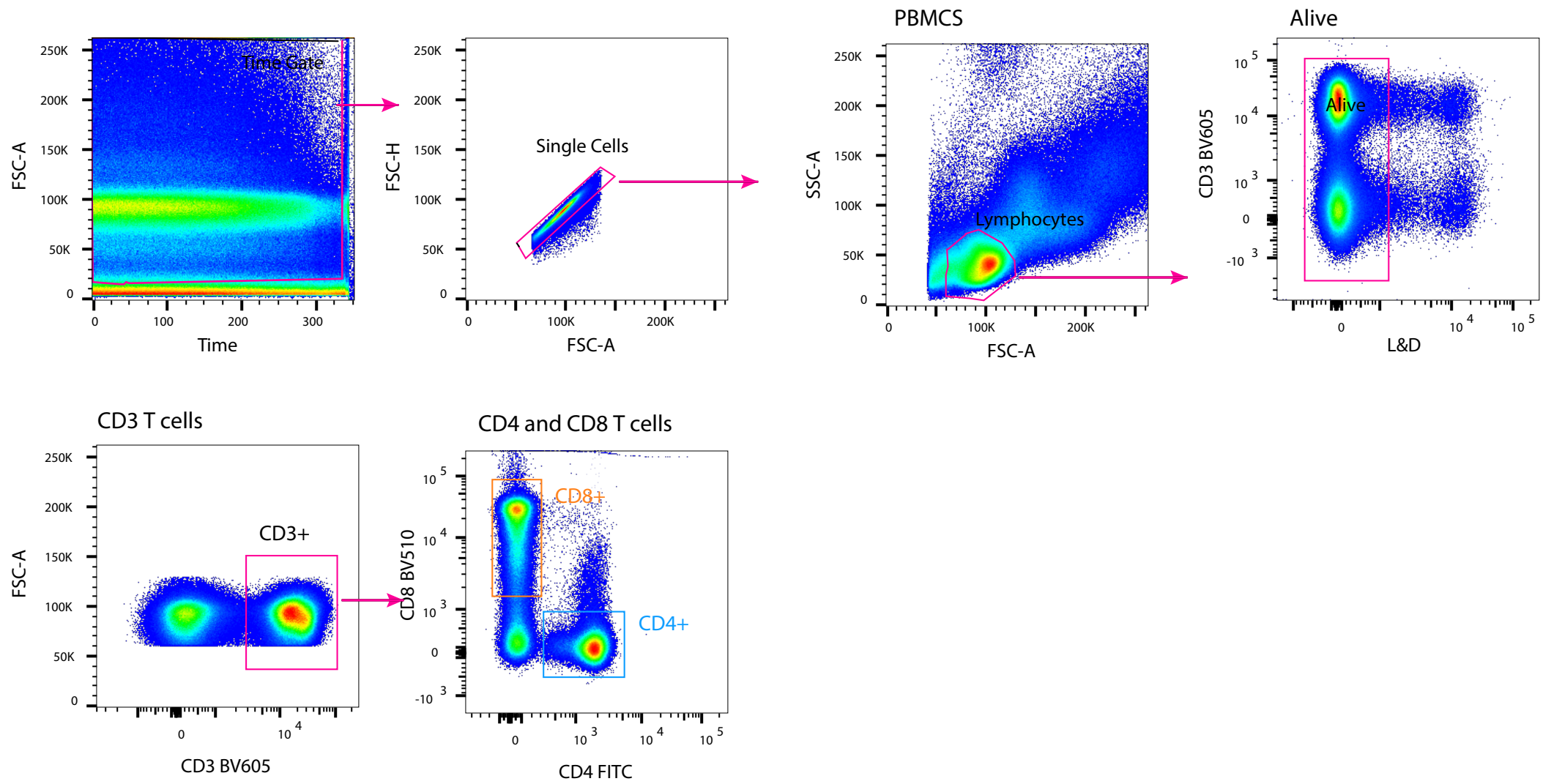

## CD4 T cells

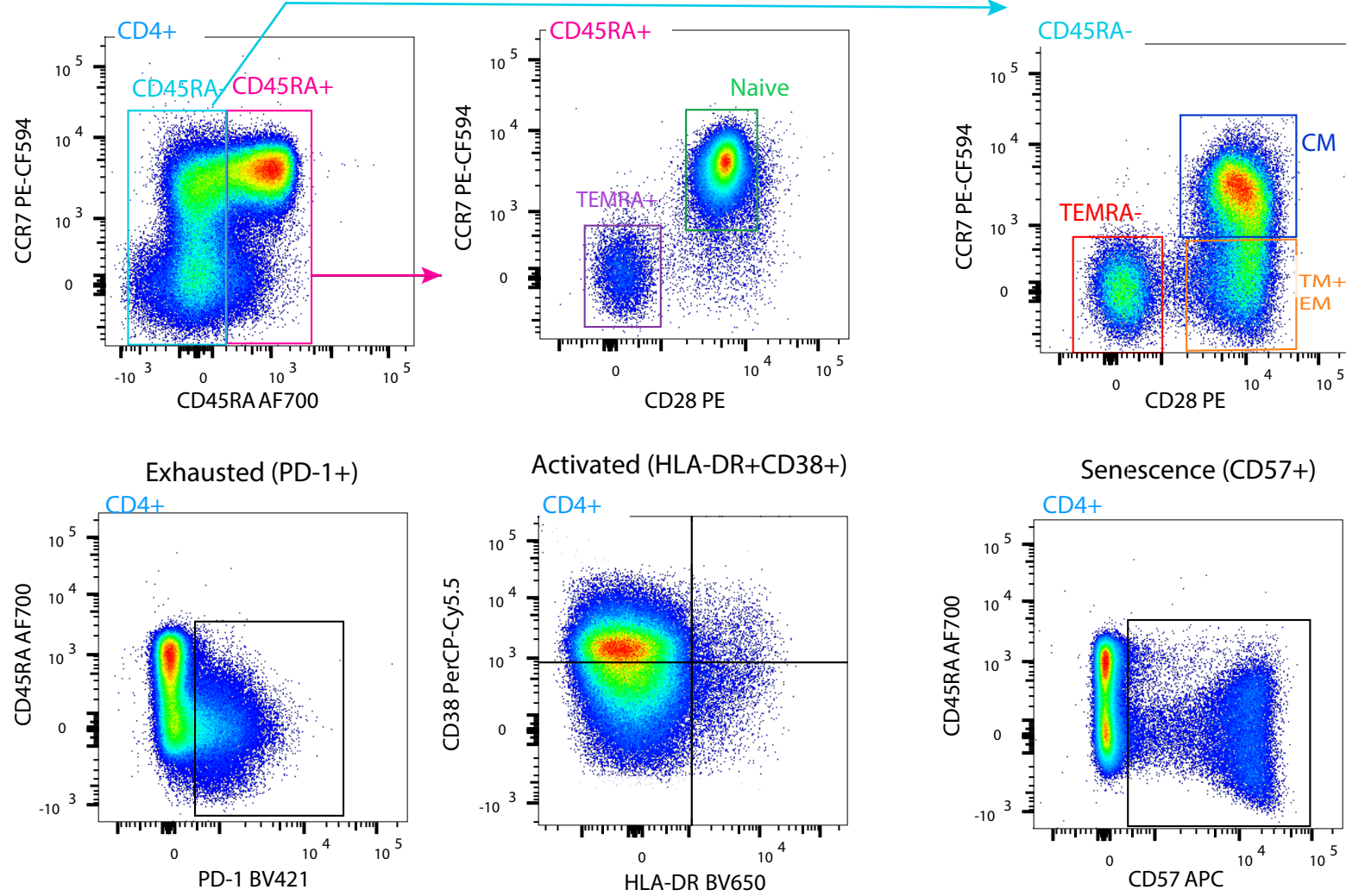

## CD8 T cells

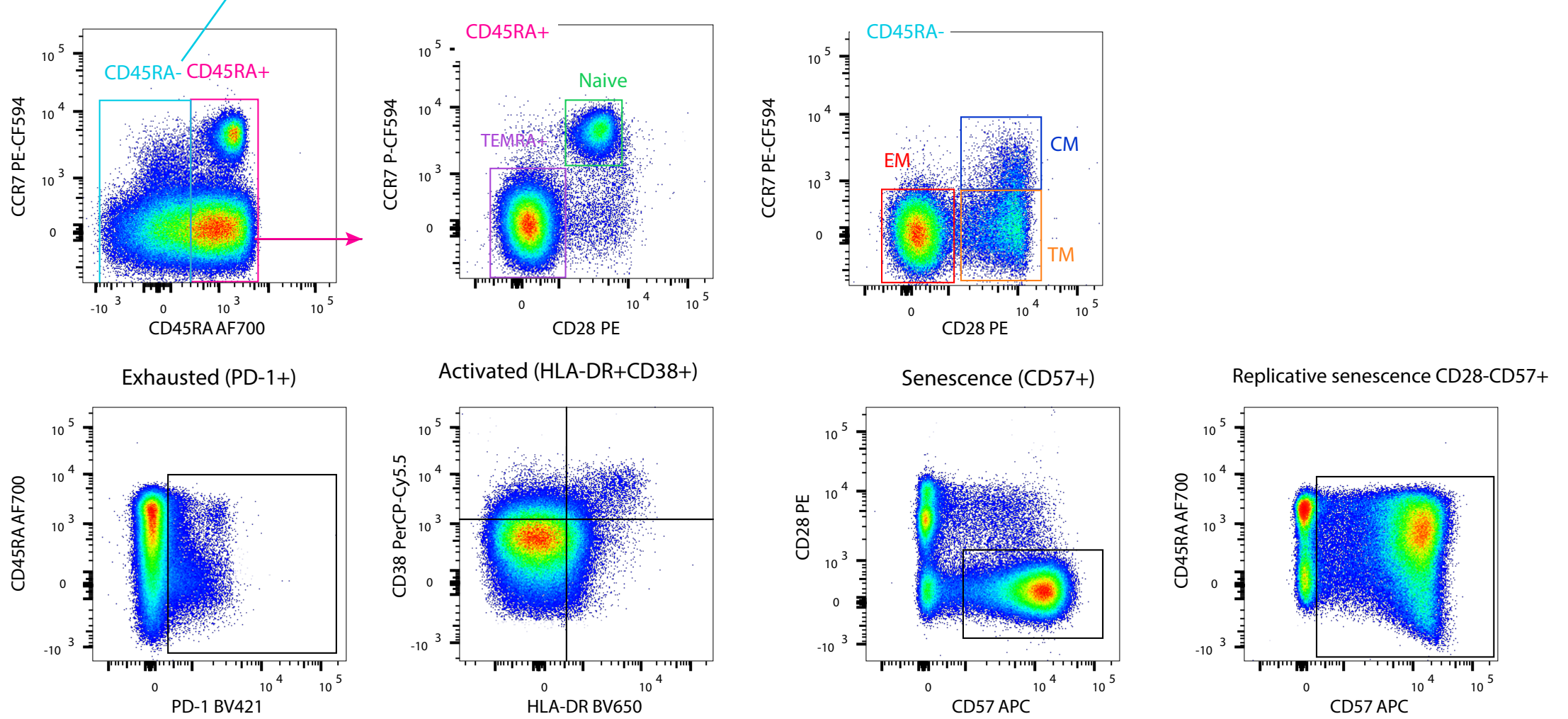

Supplement: Supplementary file 1 [file ijms-25-10930-s001.zip › Supplementary Figure s1 28092024.pdf]
